# Foreign Summary

**Published:** 1838-08-15

**Authors:** 


### FOREIGN SUMMARY.

### *Practical Remarks on Poisons, by R. D. Thomson, M. D.*

(Continued from page 264.)

11. Cocculus indicus.—*Symptoms.* Low pulse, convulsions, insensibility. *Treatment.* Emetics. Hahnemann recommends camphor as the proper antidote.

12. Coriaria.—*Symptoms.* This substance is liable to be mistaken for senna. It produces violent fits of tetanus and apoplectic coma. *Treatment.* Emetics.

13. Poisonous fungi.—*Symptoms.* Burning thirst, pain in the alimentary canal, sense of suffocation, prostration of strength, nausea, vomiting, and low pulse; tenesmus, profuse perspiration, diarrhœa, convulsive trembling, delirium, death. *Treatment.* Emetics, demulcents, oils. Acids should be carefully withheld.

14. Cantharides.—*Symptoms.* Nausea, vomiting, copious alvine evacuations, often bloody; epigastralgia, colic, pains in the hypochondria, heat in the bladder, bloody urine, priapism, pulse frequent and hard, frightful convulsions, tetanus, death. *Treatment.* Emetics, milk, mucilaginous drinks, emulsions. Groenevelt has recommended camphor to relieve the dysury and priapism.

III. Poisons producing narcotic symptoms.

Opium, hyosciamus, conium, veratrium, solanum, lactuca, belladonna, aconitum, cicuta, datura, nicotiana, colchicum, digitalis, ipecacuan, alkaline sulphurets, alcohol, carbonic oxide, carburetted hydrogen, hydrocyanic acid.

Opium.—*Symptoms.* Giddiness, stupor, nausea, vertigo, dilated and then contracted pupils, convulsions, delirium. The symptoms vary considerably, according as the dose has been large or small; but poisoning by this substance may be readily recognised by the great stupor which always accompanies its action. *Treatment.* Emetics are to be administered immediately, and the stomach-pump should be applied, if one can be easily obtained; or a flexible tube filled with water and closed at the other end with the thumb, may be introduced into the stomach and made to act like a syphon. Water may be thus introduced or removed from the stomach at will. A decoction of nut-galls should then be swallowed, in order to decompose any opium which may remain. Coffee also answers the same purpose. When the head symptoms are very severe, bleeding may be practised. Hahnemann asserts that that camphor is an antidote. The patient should be constantly roused from his state of stupor, by dragging him about the apartment and shaking him. Water should be repeatedly dashed over his head. The application of stimulants to the nostrils may also assist in removing the lethargy, as ammonia, assafœtida, &c. Artificial respiration has also been recommended.

Other narcotics.—All the substances which are usually employed in medicine as narcotics may prove deleterious when given in over-doses, if they have been prepared in a proper way; but, as usually met with in this country, the extracts and preparations of narcotic plants will have little chance of producing any very dangerous symptoms, unless they are given in enormous doses. The symptoms of poisoning from the administration of any of the narcotics in the preceding list are, giddiness, fainting, nausea, vomiting; coma, or stupor; spasms, and frequently tetanus; delirium. *Treatment.* In all cases of poison by narcotics the treatment is nearly the same, as no antidotes, that is, substances which decompose the poison, are known. The first point to attend to is, the evacuation of the poison by means of emetics; and the second object of attention is, to prevent the poison which has already been absorbed from producing such lethargy as to induce death. The same means must be adopted to effect this as were noticed under opium. *Hydrocyanic acid*, when taken in over-doses, does not produce convulsions, but a lethargy similar to that described by Shakspeare in Romeo and Juliet.

IV. Poisons producing paralytic symptoms.

Lead, mercury, manganese, chromium.

Lead.—*Symptoms.* The experiments of Mitscherlich have shown that acetate of lead does not enter into the circulation, that the salt does not produce deleterious effects, as an acetate; but that upon being introduced into the stomach it is decomposed, and forms compounds with the animal matter, some of which are soluble in water, while others are insoluble; and others again are soluble in acids. The power, therefore, of the lead to produce poisonous effects, will depend upon the state of the stomach. Poisoning by salts of lead is usually marked by derangement of the stomach, cramps in the pit of the stomach, colic, belly hard; the umbilicus much drawn in, sometimes almost touches the spine; obstinate constipation, violent pains in the legs, cold perspirations, palsy of the upper extremities, muscular emaciation, great debility. *Treatment.* The cause of the disease should be removed. Lead exists in every water which passes through lead pipes. When the lead still remains in the intestinal canal, any of the alkaline sulphates, carbonates, or phosphates, may be administered, in order to form an insoluble powder. A purgative also relieves the colic. After the full operation of the purgative, a dose of opium may be administered with good effect.

Mercury, when persevered in, or when inhaled, produces paralytic symptoms.

Manganese also, when inhaled, produces palsy—on the authority of Dr. Couper of Glasgow. *British Annals of Medicine*, vol. i.

Chrome has a similar action.

In all these cases, exposure to cool, free air, is indispensable, and the adoption of such measures as shall contribute to brace the muscular and nervous systems.—*British Medical Almanac*, 1838.

---

*Inflammation of the Testicle treated by Compression.*—Dr. Hildebrand states, that since the publication of Fricke's paper on the treatment of orchitis by compression, he had made observations on five cases, consequent on gleet which had been too suddenly arrested by the excessive use of balsam copaiba, causing sympathetic metastasis of the inflammation. In these five cases it was the left testicle which was affected. In all, Dr. H. applied pressure by means of sticking plaster, after the manner recommended by Fricke, (which we have described in a former number of this Journal,) without any preparation. In two cases only, when inflammation of the testicle was very excessive, he applied six leeches, and applied fomentations to the part for six hours, partly to encourage the bleeding, and partly to lessen the tension of the whole scrotum. In all the five cases he had seen the most extraordinary effects in the space of four or five days. The application of the compressive apparatus was not attended with the slightest inconvenience; it succeeded equally well when the patient lay in bed on his back, with his legs well separated, or when he sat on the side of the bed, or edge of a chair. The strips of plaster employed for compression were formed from the emplastrum cerussæ, and were not placed, as Fricke directs, from above downwards, but from the perinæum upwards, each strip half covering the one next to it, and proceeding thus till they came together over the pubis: he then laid a strip of adhesive plaster obliquely across the ends to secure them. From this compression, even when very tight, he had seldom seen any great pain follow. After, from twenty-four to twenty-eight hours, during which time the patients were obliged to lie in bed, with the scrotum well supported, the strips usually became loose from the diminution of the swelling. This loosened plaster was not taken off—the doing so would give great pain—but other slips laid over it, by which the pressure may be still kept up, and increased.—*Dublin Journal, from Medicinische Zeitung.*

---

*Dislocation of the Femur.*—The No. of *Guy's Hospital Reports* for April last, contains an account, by Dr. Cummins, of a case of dislocation of the femur, which, on various accounts, is worthy of notice. The subject of the case, a man about 55 years of age, whilst in a state of inebriation, fell from the road, down several feet into a field. The swelling, &c. of the parts rendered the diagnosis of the case, when the patient was first seen, difficult. By the use of antiphlogistics the tumefaction was reduced by the ninth day, when it was determined that the bone was dislocated, and extension was made with the compound pulley, and kept up during nearly an hour, while attempts were made to effect the restoration of the joint to its natural state. The want of success attending these efforts induced the surgeon to doubt the correctness of his diagnosis; and the next day Dr. Cummins was consulted. He found the symptoms as follows:—

"The right limb was shortened by fully three inches, and it could not be lengthened in any degree. The knee and toes were very much turned out; and the attempt to rotate the thigh inward produced exquisite pain, without producing any change in the position of the limb. Abduction and adduction were nearly equally difficult and painful; but flexion could, to a certain extent, be performed with less difficulty. The hip was flattened; and the trochanter major not to be discovered. There was no hard or distinct tumour on the pubes; but close below the anterior superior spine of the ilium, between it and the situation of the inferior, there was a very distinct, hard, round tumour, which could be felt moving in unison with the thigh when flexion and extension were performed. There was no crepitus, no possibility of lengthening the limb, and, of course, no successive retraction on the removal of the extending force, as takes place in fracture of the neck of the thigh bone. The tumour at the anterior superior spine was fixed in its relative position; and between its most prominent part, and the point of the spine, the distance was only a few lines, and nearly in the perpendicular, as it projected but little into the abdomen."

This condition of things satisfied Dr. Cummins that the case was one of dislocation; that the tumour, situated immediately under the anterior superior spine of the ilium, was formed by the head of the femur; and consequently that the neck of the thigh bone and trochanter major lay on the contiguous portion of the dorsum ilii, above the acetabulum; and finally, that reduction ought to be attempted by extending the joint in the direction downward and backward, raising the head of the bone and rotating the knee inward, so as to turn the head of the bone into the acetabulum.

"On the 19th, a grain of tartar emetic having been previously administered at short intervals till sickness and vertigo came on, the extension, by means of the pulleys was gradually increased. Mr. Gibson, having a towel passed under the patient's thigh and over his own shoulder, raised the head of the bone, which now left its position at the anterior superior spine, and gradually came down; and then turned the knee firmly inward, at the same time pressing it towards the opposite one. The head of the bone glided into the acetabulum without any sound or snapping—the fact being only with certainty ascertained by our finding the prominence of the trochanter returned to its proper situation; the tumour, which was formerly at the anterior superior spine, entirely removed, the knee and foot in their proper direction, and the limb of equal length with the other. The extending apparatus was removed, the patient's knees were bound together, and he was placed in bed. In a fortnight, he was sufficiently restored to be able to walk a short way out of doors, and soon entirely recovered from the effects of the injury. The reduction was effected in about twenty minutes."

---

*Spermatocele, or Varicocele of the Spermatic Cord.*—The same number of *Guy's Hospital Reports* contains the following remarks on this subject, by Sir Astley Cooper.

In general, this affection produces only inconvenience to the patient, and the plan of treatment then consists in supporting the part; and Sir Astley recommends that this be "effected by applying a suspensory sling, with two tapes sufficiently long to encircle the abdomen. The sling receives the scrotum and testis; and the tapes, passed around the abdomen, and tied in front, secure the parts in an elevated position. No straps should be placed beneath, to pass between the thighs, as they draw back, rather than elevate, the scrotum and swelling.

"As the parts should be kept as cool as possible, the material of the sling should be an open *silk net*, which allows the escape of heat, and prevents a relaxing perspiration. From this support the patient derives great relief; and the application of an evaporating lotion of spirits-of-wine and water relieves him still more. A very good lotion for this purpose consists of aluminis ʒi. aquæ ℥xi. spiritus vini ℥i.; but the lotion should be as much as possible devoid of smell, as it leads to the suspicion of some infirmity.

"Washing two or three times a day with cold water, with salt dissolved in it, is useful; and the employment of the shower-bath, or common cold-bath, by constringing the scrotum, prevents the increase of the complaint.

"The dress should be as light as possible, to prevent the production of superfluous heat, and to permit its escape; and all tight dress around the abdomen is to be avoided, to allow of the free return of the venous blood from the testis. Still, however, these means leave the patient with the badge of his infirmity, from his continuing to wear his bandage; and attempts have been made to relieve him, by exciting inflammation and thickening of the scrotum, and thus to render it a better support to the testes. I have applied the pyroligneous acid for this purpose; but the pain which it excited was severe, and the good effect only temporary. I have also employed blisters with the same view, and with the same effect.

"It has been advised to draw the scrotum through a ring, and fix it there, the person continuing to wear it; but, as it may be readily believed, this has no advantage over the use of the sling-support; and is a much greater annoyance to the patient's feelings, either than the disease itself, or the bandage which he is usually called upon to wear."

There are cases, however, in which this complaint produces so much pain and distress, as to render it absolutely necessary to do something more than is generally advised. Sir Astley has seen, in the course of his practice, many persons suffer so severely in mind and body from it, that they would readily submit to any operation which was not attended with danger to life, to obtain relief. As to tying the veins of the spermatic cord—from what he has seen of the dangerous and destructive effect of exciting inflammation in veins—he should never propose it; nor does he think, if it were not dangerous, that it is founded on proper principles. But in his Work on the Testis, published in the year 1830, he has advised the removal of a portion of the scrotum, in the following words:—

"*The removal of a portion of the scrotum will lead to a diminution of the veins of the spermatic cord; and it is an operation, in an extreme enlargement accompanied with pain, which might be tried with perfect safety, and is very likely to succeed.*"

He had, at that time, never performed the operation, and he therefore spoke of the probability of success only; but, aware of its being free from danger, and seeing that it would render the remaining portion of the scrotum a natural bandage, and that a great degree of relaxation of the scrotum also attended this complaint, and that such relaxed portion might be safely and effectually removed, he determined to take some opportunity of performing the operation.

"Beside the advantage of making the scrotum, in its lessened state, a means of support, he observes, it must naturally occur, that the adhesion, excited by the operation of the fascia, which covers the cremaster, to the surrounding parts, would produce a permanent support, and render a suspensory bandage unnecessary. It might be thought a painful operation; but it is not so, nor does it excite constitutional irritation.

"The mode of performing it is as follows:—The patient being placed in the recumbent posture, the relaxed scrotum is drawn between the fingers; the testis is to be raised to the external ring by an assistant; and then the portion of the scrotum is removed by the knife or knife-scissors;—but I prefer the former. Any artery of the scrotum which bleeds is to be tied; and a suture is then made, to bring the edges of the diminished scrotum together. The patient should be kept for a few hours in the recumbent posture, to prevent any tendency to bleeding; and then a suspensory bag is to be applied, to press the testis upwards, and to glue the scrotum to the surface.

"The only difficulty, in the operation of removing the scrotum by excision, is in ascertaining the proper quantity to be removed; but it adds but little to the pain if a second portion be taken away, if the first does not make sufficient pressure on the spermatic cord. It is of no use to remove a small portion of the scrotum, for from doing this I have failed. When the wound has healed, the varicocele is lessened, but not always entirely removed; but the pain and distressing sensations cease, if sufficient of the scrotum be removed.

"In making the suture in the scrotum, its lower part is to be brought up towards the abdominal ring, to raise and support the testis; as does the suspensory sling when it is worn."

The following cases are given in which this operation was performed.

Case I. Mr. Rees, surgeon, of Blackfriars Road, sent me a patient of his, who had a large varicocele on the left side, with a very relaxed scrotum. He suffered severely from uneasiness in the spermatic cord and in the loins, a sense of weight and oppression in the region of the stomach, and excessive mental depression. On the 18th of February, 1831, I removed a large portion of the scrotum; and exposed the fascia covering the cremaster, and the testis in its envelopes. By three sutures, the edges of the scrotum were approximated, and the wound quickly healed; and he, on the 3d of March afterwards, quitted London,"

This gentleman was 32 years of age. The portion of scrotum removed, when extended, measured four inches in length; and in breadth, in the middle, two inches and a half. He left London quite well, and some time afterwards Sir Astley learned from Mr. Webster that the patient was able to ride fifty miles a day, without inconvenience.

"Case II. Mr. S——, aged 20, has had a spermatocele three years and a half, attended with a great sense of uneasiness in the part, and a dull heavy pain in the spermatic cord and loin on that side. My assistant, Mr. Balderson, held the scrotum between his fingers; and I removed all that could be easily elevated from the testis and its coverings, which are necessarily exposed in the operation. I then brought the integuments together by sutures, so as to close the wound completely; but I previously secured some small bleeding arteries. He was ordered to keep himself cool, and to remain in the recumbent posture; and the part was placed in a suspensory sling: however, the next morning he went down to breakfast; but this imprudence did not prevent his quick recovery from the operation, with the result of which he was highly pleased. The varicose veins are greatly reduced: the coverings of the testis adhere to the upper part of the scrotum. He soon gave up the use of the sling-support; and lost the pain in the spermatic cord and loins, which he had previously sustained."

"Case III. H. B., aged 18 years, had a spermatocele upon the left side, from the age of fourteen. At fifteen he fell across an iron bar, which greatly hurt him; and he thought the complaint had quickly increased after that time. He suffered much from pain in the testis, more especially in walking, and from uneasiness in the groin, spermatic cord, and the spinous process of the ilium and loins. He consulted several medical men, who told him his complaint was a hernia. But he was then recommended to Mr. Taunton, in Hatton Garden, who informed him it was a varicocele: and the scrotum was directed to be supported, and an evaporating lotion to be used.

"On July 20, 1837, I removed a large portion of the relaxed scrotum which covered the swelling, in the presence of Mr. James Babington; secured some small arteries; and then used four sutures, to approximate the edges of the scrotum. He was sent from my house, in a coach to Chelsea, after the operation, and the scrotum very soon healed, and the uneasy sensation in the part vanished."

"Case IV. Mr. John K——, aged 25, four months ago found the scrotum enlarged on the left side, which occasioned pain in the part, which darted upwards to the external abdominal ring. It gradually increased, until it was three times larger than the right side of the scrotum, became more painful, and occasioned much depression of spirits. On the 15th of October, 1837, I removed a portion of the scrotum, by passing a needle and thread through it in three different places, and cutting away the scrotum beyond them. This plan did not facilitate the operation, and made the tying of the arteries more difficult; but it succeeded in relieving the disease."

In one case Sir Astley raised the scrotum, and placed a ligature around the part which he designed to remove, drawing the thread quite tight: but it produced a great deal of pain; the part sloughed with considerable constitutional irritation, and after a great length of time, and with more suffering than the complaint justifies.

It must be distinctly understood that the removal of a portion of the scrotum is recommended in those cases only of spermatocele, in which the patient suffers great local pain; in cases in which he is most urgent to have the swelling and deformity of the part removed; and more especially in those instances in which the function of digestion suffers, and there is a great degree of nervousness and mental depression.

